# A Downlink and Uplink Alignment Scheme for Power Saving in IEEE 802.16 Protocol

**DOI:** 10.1155/2014/217973

**Published:** 2014-01-12

**Authors:** Jenhui Chen, Woei-Hwa Tarn, Jiann-Der Lee

**Affiliations:** ^1^Department of Computer Science and Information Engineering, School of Electrical and Computer Engineering, College of Engineering, Chang Gung University, Kweishan, Taoyuan 33302, Taiwan; ^2^Department of Electrical Engineering, School of Electrical and Computer Engineering, College of Engineering, Chang Gung University, Kweishan, Taoyuan 33302, Taiwan

## Abstract

This study shows the problem of power saving mechanism (PSM) that sleep intervals of uplink (UL) connections do not synchronize with sleep intervals of downlink (DL) connections. That is, the energy of a mobile station (MS) is not *really* saved if the DL connections are in the sleep mode while the UL connections are in normal mode, and vice versa. To avoid the asynchronism of power saving (PS) between UL and DL connections, we invent a mechanism of DL connections regulating UL connections, called *DL and UL Alignment* (DUAL) scheme, to improve the energy efficiency for PS. Considering that the buffer size of MS is limited, DUAL uses the mean packet arrival rate of UL *λ*_*u*_ and a relatively safe threshold of buffer size *Q*_*T*_ as the parameters to estimate the maximum allowable waiting time to align the UL with the DL connections. To analyze the performance of DUAL, a system model of PS is proposed to evaluate the performance of DUAL under different conditions. The correctness of performance analysis of DUAL is validated by using simulation with realistic parameters. Numerical experiments show that DUAL improves the energy conservation significantly when UL traffic is greater than DL traffic.

## 1. Introduction

In the IEEE 802.16 system, a mobile station (MS) registered with a base station (BS) can be either in the *normal mode* or in the *sleep mode*. MSs, in the normal mode, can send or receive data according to the scheduling announcement issued from the serving BS (SBS). On the contrary, in the sleep mode, MSs are absent from the air interface of its SBS for prenegotiated time intervals and conserve energy by powering down during sleep windows. The IEEE 802.16-2009 standard [1] (merging 802.16-2004 and 802.16e/f/g/i) provides three different power saving classes (PSCs) in power saving mechanism (PSM) for sleep mode usage. The PSC type I is designed for connections of best effort (BE) and nonreal-time (NRT) traffic, PSC type II is recommended for unsolicited grant service (UGS) and real-time variable bit rate (RT-VBR) connections, and PSC type III is for multicast and management connections, respectively.

For the PSC type I, the sleep mode comprises two kinds of operational windows (time intervals), the sleep window and the listening window. If an MS wishes to enter the sleep mode from the normal mode, the MS shall inform its SBS by a sleep request message (MOB_SLP-REQ) and obtain the SBS's approval through a sleep response message (MOB_SLP-RSP) with parameters initial-sleep window *T*_min_, maximum sleep window *T*_max_, and listening window *T*_*L*_. After receiving the MOB_SLP-RSP message, the MS then enters the sleep mode.

The MS turns its transceiver off during sleep windows in order to reduce the energy consumption. In the sleep mode, the MS will wake up in any of the following circumstances: (a) the positive traffic indication (MOB_TRF-IND) management message issued from the SBS in the listening window if there is traffic in the downlink (DL) connection for it; (b) the MS has uplink (UL) traffic itself (the MS has to wake up immediately when UL traffic arrives).

Many PSMs had been broadly studied such as maximum unavailability interval (MUI) algorithm [2], adaptive power saving mechanism (APSM) [3], scheduled power-saving mechanism (SPSM) [4], performance analysis of power saving for heterogeneous traffic [5], power saving studies based on QoS constraints [6], statistical sleep window control (SSWC) approach [7], and an analytical modeling for power saving [8] recently. However aforementioned schemes only consider the power saving (PS) on one way connection and neglect the activity of reverse connections. The lack of consideration of *two-way connections* together for PS may lead to a worst case that one way connections are in sleep mode while the reverse way connections are in normal mode. Practically, the MS cannot enter sleep mode if the status of any connection is in normal mode. Depending on this symptom, we propose a DL and UL alignment (DUAL) scheme to solve this problem as well as analyze the performance of DUAL in this paper. Specifically, the contributions of this paper are as follows:

- the problem of DL and UL sleep intervals (windows) being not synchronous is studied and first solved by using the proposed DUAL scheme;
- a *T*_*E*_ estimation function (presented in Section 3) for extending the sleep duration of UL by aligning UL sleep interval with DL's is investigated.

The rest of this paper is organized as follows. In Section 2, we present the system model of the PSM. The detailed mechanism of DUAL is shown in Section 3. Additionally, we also illustrate how DUAL works in an example in this section. In Section 4, we present the energy consumption analyses for DUAL as well as the legacy IEEE 802.16 PSM. The correctness of the energy consumption analysis is validated by simulations in Section 5. The detailed numerical experiments are also given in Section 5. Finally, we conclude the benefits of DUAL and its applications in Section 6.

## 2. System Model

In the IEEE 802.16-2009 standard, if an MS does not have any UL and DL traffic persisting for a duration time *T*_0_, it will enter the sleep mode. In the sleep mode, the PSC type I follows a *binary exponential sleep window* mechanism which is expressed as

(1) $$\begin{array}{c} T_{n}=\{\begin{array}{cc} T_{\min}, & n=1,\\ \min\left(2^{n-1}T_{\min},T_{\max}\right), & n>1, \end{array} \end{array}$$

where *T*_*n*_ represents the *n*th sleep window time and *T*_min_ and *T*_max_ are the minimum and maximum value of the sleep window. The duration of the *n*th sleep cycle, denoted by *W*_*n*_, consists of one variable sleep window *T*_*n*_ and one fixed listening window *T*_*L*_ and is expressed as

(2) $$\begin{array}{c} W_{n}=T_{n}+T_{L}. \end{array}$$

Then,

(3) $$\begin{array}{c} Z_{n-1}=\overset{n-1}{\underset{i=1}{\sum }}W_{i}=\overset{n-1}{\underset{i=1}{\sum }}\left(T_{i}+T_{L}\right) \end{array}$$

is the duration time from the start time of the sleep mode to the end of *W*_*n*−1_.

Assume that the DL (from the SBS to the MS) and the UL (from the MS to the SBS) traffic for any MS follows the Poisson process with mean packet arrival rates of DL *λ*_*d*_ and UL *λ*_*u*_, respectively. Let *λ* denote the sum of DL and UL packet arrival rates to an MS and let *λ* = *λ*_*d*_ + *λ*_*u*_ [9]. The probability of no arrivals in time duration *T* is written as *p*_0_(*T*) = *e* ^−Λ*T*^, where Λ can be either *λ*_*u*_ or *λ*_*d*_, for example, *e* ^−*λ*_*u*_*T*^ represents the probability of no packet coming to the MS in UL in time duration *T*.

## 3. DL and UL Alignment (DUAL) Scheme

In the legacy IEEE 802.16 PSM, when a UL traffic arrives, the MS will wake up automatically to contend the bandwidth for transmission of the UL traffic. The legacy IEEE 802.16 PSM doesn't provide the PS function for UL traffic because UL transmissions are only triggered by the MS. Therefore, it cannot be involved in the PSM of the IEEE 802.16 standard [1].

To tackle the asynchronous problem among UL and DL connections and increase the duration of actual interval in sleep mode, DUAL takes the advantage of temporally delaying these UL packets of the MS in the buffer if no traffic happens in the opposite DL direction. To enable this capability of two directional connections alignment, we investigate an estimation function to determine a duration time *T*_*E*_ for temporally storing the UL packets with the consideration of not leading to the buffer overflowing problem. Let *Q*_*T*_ be the buffering threshold which is a relatively safe quantity for packets to be stored in the SBS buffer temporarily. The value of *Q*_*T*_ varies depending on the size of buffer the MS has. Let *Q* be the buffer size of the MS, then we have

(4) $$\begin{array}{c} Q_{T}=\alpha Q, \end{array}$$

where *α* = [0,1], *α* ∈ **R**; for example, *α* = 0.8, is the safely buffering coefficient. We note that the value of *α* can be different and set depending on different requirements or situations. After setting *Q*_*T*_, according to *λ*_*u*_ and the mean packet size *L* of arrivals, we can estimate the longest tolerable period of time for temporarily storing by

(5) $$\begin{array}{c} T_{E}=\frac{Q_{T}}{\lambda_{u}L}=\frac{\alpha Q}{\lambda_{u}L}. \end{array}$$

**Figure 1:**
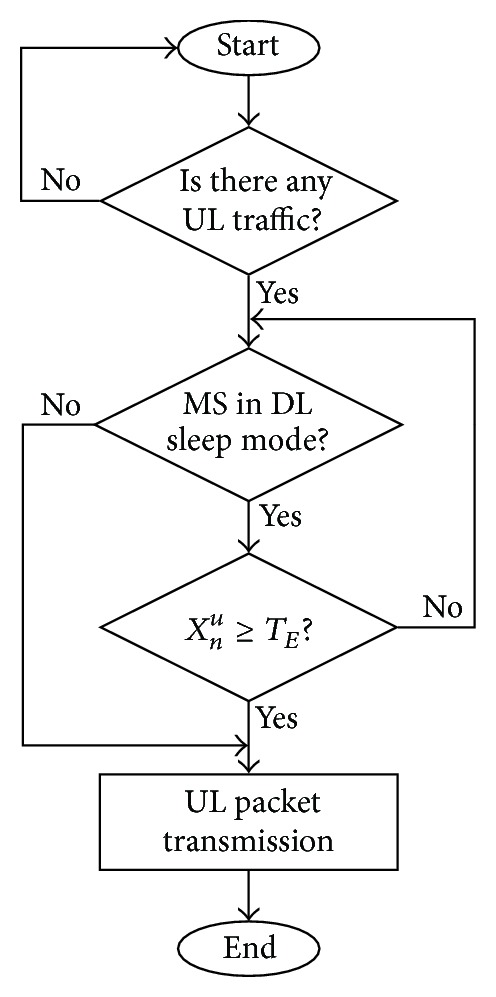
The algorithm of DUAL scheme.

Based on the derived *T*_*E*_, DUAL is designed as the following logic. If there is any UL traffic for the MS, the MS will check the condition whether it is in the sleep mode or not. If the MS is not in the sleep mode, the MS contends the bandwidth for transmission of packets immediately and does not consider the DL traffic. If the MS is in the sleep mode, the MS stores the UL packets in the buffer temporally and stays in the sleep mode until the notification of the positive MOB_TRF-IND. In this case, there will be two possible consequences. The first possible consequence is that the MS receives the positive MOB_TRF-IND issued from its SBS before it reaches the time limitation of *T*_*E*_. The second possible consequence is that there is no DL traffic until it reaches *T*_*E*_. In either consequence, the MS will wake up from sleep mode and contend the bandwidth for transmitting the buffered packets. The algorithm of DUAL is given in Figure 1.

**Figure 2:**
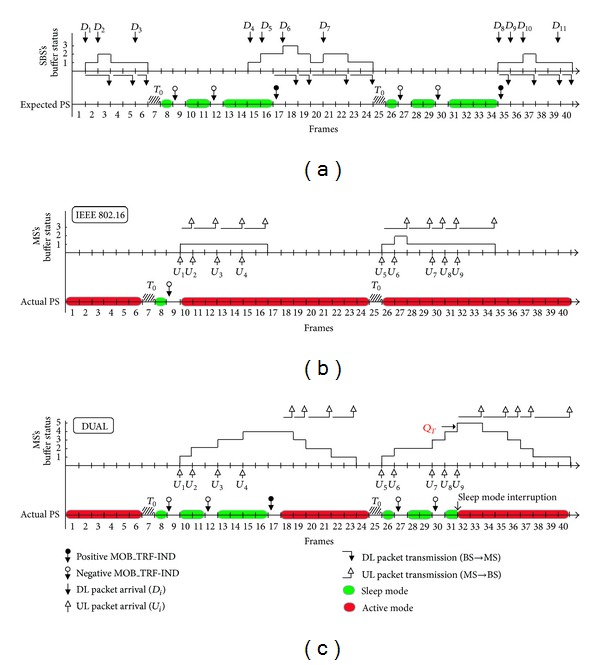
An illustration of the buffer status of SBS and MSs in time diagram: (a) the SBS side; (b) the MS side in the IEEE 802.16-2009 case; (c) the MS side in DUAL case.

Figure 2 illustrates the buffer status of the SBS and the MSs by comparing with the legacy IEEE 802.16 PSM (Figure 2(b)) and DUAL (Figure 2(c)).  Figure 2(a) shows the status of the DL packet arrivals and the variation of the SBS's buffer. The time of the diagram is divided into frames. For simplicity, we assume the length of *T*_min_, *T*_*L*_, and *T*_0_ to be one frame. The DL and UL packet arrivals are denoted as *D*_*i*_ (solid arrow), *i* = 1,2,… and *U*_*i*_ (hollow arrow), respectively. In this example, the packet length is variant.  Figure 2(a) shows the packet arrivals (DL packet arrivals) in the SBS. The PS durations (colored green in the time axis) are determined according to the situation of DL packet arrivals. The expected PS duration is calculated by the PSC type I of the IEEE 802.16 PSM. Therefore, without considering the UL, the PS durations will be same as shown in Figure 2(a).

However, Figure 2(b) shows the actual PS situation that happened in the MS by considering the actual UL packet arrivals. We can see from Figure 2(b) that the MS uses the transceiver's power to transmit or receive packets (UL or DL) colored with red ink in the time axis. We use an extreme example that the UL traffic is almost interleaved with the DL traffic to illustrate the PS problems. In Figure 2(a), the MS enters the sleep mode from frame number 8 to frame number 16 in DL connection. However, when the UL traffic is considered, the MS wakes up earlier in frame number 10 due to the arrivals of UL traffic. In the same way, the MS does not enter the sleep mode from frame number 26 to frame number 34 since the UL packets arrive. We can see the problem that the PSM of the IEEE 802.16 only considers the DL arrivals and does not consider the DL arrivals. As a result, the energy of the MS is not really saved as described in the PSM of the IEEE 802.16 standard.

Figure 2(c) shows the actual PS situation by adopting the DUAL scheme. When UL packets *U*_1_, *U*_2_, *U*_3_, and *U*_4_ arrive, they are temporally stored in the MS's buffer. The UL connection does not interrupt the sleep mode of the DL because DUAL postpones these UL packets until the DL packets arrive. Thus, the MS gets a sleep interval from frame number 8 to frame number 16 until the arrival of the positive MOB_TRF-IND. Figure 2(c) also shows the case that the MS interrupts the sleep mode when the quantity of temporarily stored UL packets reaches the threshold *Q*_*T*_ at frame number 32. This mechanism prevents the MS's buffer from the consequence of buffer overflowing.

## 4. Performance Analysis

### 4.1. DUAL Energy Consumption

**Figure 3:**
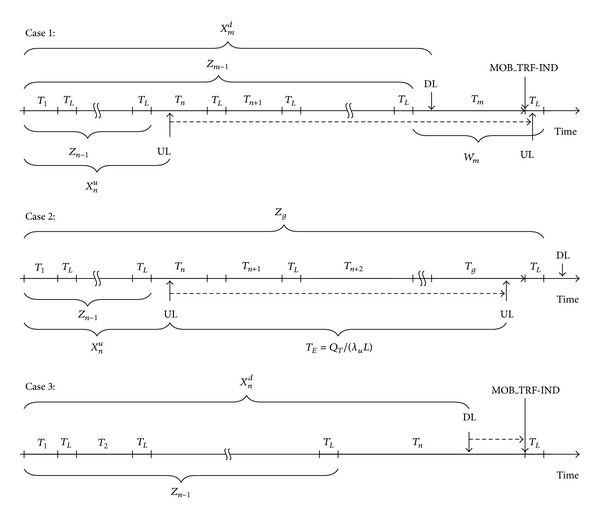
An illustration of the three cases of DUAL.

According to the actual condition, we classify the PS operations for DUAL into three cases as illustrated in Figure 3. Let *E*_*n*_^(*i*)^ and *p*_*n*_^(*i*)^ represent the consumed energy and corresponding probability in the case *i*, *i* = 1,2, 3.

**Case 1**

The first UL packet arrives during *T*_*n*_ and later a DL packet arrives during *T*_*m*_. In order to synchronize the DL and UL connections and save more energy, the MS temporarily buffers the UL packet until the DL packet arrives.

**Case 2**

UL packets arrive during *T*_*n*_ and are temporally buffered in MS's queue. The buffered size of UL reaches the relatively safe threshold *Q*_*T*_ before the arrival of DL's packets; the MS is waked up from the sleep mode.

**Case 3**

DL packets arrive during *T*_*n*_ and are temporarily buffered in the BS until the end of *T*_*n*_. Meanwhile there is no UL packet that arrives in *T*_*n*_.

The details of these three kinds of cases are separately discussed below.

*Case 1*. As shown in Figure 3, the case assumes that the first packet of UL arrives during *T*_*n*_ or the preceding *T*_*L*_ of *T*_*n*_ (i.e., *T*_*L*_ of *W*_*n*−1_). Let *X*_*n*_^*u*^ be the random variable, which is the persistent duration from the start time of the sleep mode until the coming of UL arrivals in the preceding *T*_*L*_ of *W*_*n*_ or in *T*_*n*_. In the same way, let *X*_*m*_^*d*^ denote the random variable of the coming of DL arrivals in the preceding *T*_*L*_ of sleep cycle *W*_*m*−1_ or in sleep window *T*_*m*_, which is subject to *n* ≤ *m* and *Z*_*m*−1_ + *T*_*m*_ < *Z*_*n*−1_ − *T*_*L*_ + *T*_*E*_. In this case, the first DL arrival could be in *T*_*n*_, *T*_*n*+1_,…, *T*_*k*_, where *T*_*k*_ is the farthest sleep window that the MS can reach without exceeding the upper-bound buffering size *Q*_*T*_. The length of maximal sleeping duration follows the condition

(6) $$\begin{array}{c} T_{L}+2T_{n}+T_{L}+2^{2}T_{n}+\cdots +T_{L}+2^{k-n}T_{n}<T_{E}. \end{array}$$

Finishing the inequality (6), it is rewritten as

(7) $$\begin{array}{c} \left(k-n\right)T_{L}+2\left(2^{k-n}-1\right)T_{n}<T_{E}. \end{array}$$

Because (*k* − *n*)*T*_*L*_ ≪ 2(2^*k*−*n*^ − 1)*T*_*n*_, (*k* − *n*)*T*_*L*_ can be ignored and

(8) $$\begin{array}{c} 2\left(2^{k-n}-1\right)T_{n}<T_{E}. \end{array}$$

Rearranging (8), it becomes

(9) $$\begin{array}{c} 2^{k-n+1}T_{n}<T_{E}+2T_{n}. \end{array}$$

Apply log_2_ on both sides of (9) for *k*; then

(10) $$\begin{array}{c} k<n-1+\log_{2}\left(\frac{T_{E}}{T_{n}}+2\right). \end{array}$$

Because *k* = 0,1,… is a positive integer, the maximal value of *k* can be obtained by

(11) $$\begin{array}{c} k=\left\lfloor n-1+\log_{2}\left(\frac{Q_{T}}{\lambda_{u}LT_{n}}+2\right)\right\rfloor . \end{array}$$

From (11), we know that *k* − *n* is greater as *T*_*n*_ is smaller. It means that the MS can postpone UL packets longer and save more energy if *n* is smaller. Inversely, *k* − *n* is smaller as *T*_*n*_ is greater. That means that MS cannot postpone UL packets too long.

Because the random variable *m* = *n*, *n* + 1,…, *k*, we have to determine the appearing probability of each value of *T*_*m*_ by given *n*. The appearing probability of *T*_*m*_ denoted as *P*_*nm*_^(1)^, where the suffixes *n* and *m* mean the arrivals of UL in *T*_*n*_ and the arrivals of DL in *T*_*m*_, is calculated as

(12) pnm(1)=Pr{UL arrival in TL+Tn}·Pr{DL arrival in TL+Tm}=Pr{Zn−1−TL<Xnu≤Zn−1+Tn}·Pr{Zm−1−TL<Xmd≤Zm−1+Tm}=(∫0Zn−1+Tnλue−λuxdx−∫0Zn−1−TLλue−λuxdx)·(∫0Zm−1+Tmλde−λdxdx−∫0Zm−1−TLλde−λdxdx)=e−λu(Zn−1−TL)(1−e−λuWn)·e−λd(Zm−1−TL)(1−e−λdWm),

which is subject to

(13) $$\begin{array}{c} Z_{m-1}+T_{m}<X_{n}^{u}+T_{E}, \end{array}$$

where *X*_*n*_^*u*^ = (*Z*_*n*−1_ − *T*_*L*_, *Z*_*n*−1_ + *T*_*n*_]. Then the energy consumption of the arrival of UL in *T*_*n*_ and the arrival of DL in *T*_*m*_, denoted as *E*_*nm*_, can be obtained by

(14) $$\begin{array}{c} E_{nm}=\overset{m}{\underset{i=1}{\sum }}T_{i}E_{S}+\left(m-1\right)T_{L}E_{L}, \end{array}$$

where *E*_*S*_ represents the energy consumption per time unit in sleep window and *E*_*L*_ represents the energy consumption per time unit in listening window. According to (12) and (14), we can obtain the energy consumption of Case 1, *E*_*n*_^(1)^:

(15) En(1)=pnn(1)Enn+pn(n+1)(1)En(n+1)+⋯+pnk(1)Enk=∑i=nkpni(1)Eni,

where *k* is given in (11).

*Case 2*. As shown in Figure 3, Case 2 assumes that the arrival time of DL *X*_*m*_^*d*^ exceeds the limited time *T*_*E*_ since the first arrival of UL in *T*_*n*_, that is, *X*_*n*_^*u*^ + *T*_*E*_ < *X*_*m*_^*d*^. In the case, the time which the MS is enforced to wake up is $\bar{X_{n}^{u}}+T_{E}$, where $\bar{X_{n}^{u}}$ is the mean time that the first arrival of UL occurs. According to (10), the MS will wake up in *T*_*g*_ (the *g*th sleep window), where *g* = ⌈*n* − 1 + log_2_((*Q*_*T*_/*λ*_*u*_ *LT*_*n*_) + 2)⌉. The mean time of *X*_*n*_^*u*^ can be obtained by

(16) Xnu¯=E[Xnu ∣ Zn−1−TL<Xnu≤Zn−TL]=∫Zn−1−TLZn−1+Tnxλue−λuxdx(∫Zn−1−TLZn−1+Tnλue−λuxdx)−1=e−λuZn−1[eλuTL(Zn−1−TL+1λu)    −e−λuTn(Zn−1+Tn+1λu)]·{e−λu(Zn−1−TL)−e−λu(Zn−1+Tn)}−1.

Because *X*_*n*_^*u*^ + *T*_*E*_ falls in the *g*th sleep window, that is, *Z*_*g*−1_ < *X*_*n*_^*u*^ + *T*_*E*_ ≤ *Z*_*g*_ − *T*_*L*_, the energy consumption of Case 2 is calculated as

(17) $$\begin{array}{c} E_{n}^{\left(2\right)}=\overset{g-1}{\underset{i=1}{\sum }}T_{i}E_{S}+\left(g-1\right)T_{L}E_{L}+\left(\bar{X_{n}^{u}}+T_{E}-Z_{g-1}\right)E_{S}. \end{array}$$

We note that the enforced wake up time of the MS may happen in the *T*_*L*_ of *W*_*g*_. In such a case, the MS must be awake and the energy in *T*_*L*_ does not need to be counted. The appearing probability of Case 2 can be obtained by

(18) png(2)=Pr{UL arrival in TL+Tn}·Pr{no DL arrival in Xnu¯+TE}=Pr{Zn−1−TL<Xnu≤Zn−1+Tn}e−λd(Xnu¯+TE)=(∫0Zn−1+Tnλue−λuxdx−∫0Zn−1−TLλue−λuxdx)·e−λd(Xnu¯+TE)=e−λu(Zn−1−TL)(1−e−λuWn)e−λd(Xnu¯+TE).

*Case 3*. In Case 3, the DL packets arrive in *T*_*n*_ and are temporarily queued in the SBS. Because the MS will wake up in the *n*th listening window, the energy consumption is

(19) $$\begin{array}{c} E_{n}^{\left(3\right)}=\overset{n}{\underset{i=1}{\sum }}T_{i}E_{S}+\left(n-1\right)T_{L}E_{L}. \end{array}$$

The probability of Case 3 can be obtained from

(20) pnn(3)=Pr{DL arrival in Tn}·Pr{no UL arrival in Tn}=Pr{Zn−1<Xnd≤Zn−1+Tn}·e−λu(Zn−1+Tn)=(∫0Zn−1+Tnλde−λdxdx−∫0Zn−1λde−λdxdx)·e−λu(Zn−1+Tn)=e−λZn−1(1−e−λdTn)e−λuTn.

According to (15), (17), (18), (19), and (20), we can get the energy consumption of DUAL in sleep mode as

(21) $$\begin{array}{c} E_{S_{D}}=\overset{\infty }{\underset{n=1}{\sum }}\left(E_{n}^{\left(1\right)}+E_{n}^{\left(2\right)}p_{ng}^{\left(2\right)}+E_{n}^{\left(3\right)}p_{nn}^{\left(3\right)}\right). \end{array}$$

Because the MS switches between the normal mode and the sleep mode (the listening window is considered as the sleep mode), the normal mode energy consumption has to be further calculated. From (21) we can obtain the average energy consumption in sleep mode. From (12), (18), and (20), we can obtain the probability that the MS gets in the sleep mode *P*_*S*_*D*__ and be expressed as

(22) $$\begin{array}{c} P_{S_{D}}=\overset{\infty }{\underset{n=1}{\sum }}\left(\overset{k}{\underset{i=n}{\sum }}p_{ni}^{\left(1\right)}+p_{n}^{\left(2\right)}+p_{n}^{\left(3\right)}\right). \end{array}$$

Then, the energy consumption in normal mode can be derived as

(23) $$\begin{array}{c} E_{N_{D}}=\left(1-P_{S_{D}}\right)E_{A}, \end{array}$$

where *E*_*A*_ is the energy consumption per unit time in normal mode. Combining (21) and (23), the average energy consumption per unit time of the DUAL can be obtained as

(24) $$\begin{array}{c} \bar{E_{D}}=\left(1-P_{S_{D}}\right)E_{A}+E_{S_{D}}. \end{array}$$

### 4.2. Legacy IEEE 802.16 Energy Consumption

Zhang and Fujise [10] had investigated the energy consumption in sleep mode of the IEEE 802.16 PSM. They classified the behavior of the IEEE 802.16 PSM into three cases and derived the consumed energy *E*_*n*_^(*i*)^ and its corresponding probability *p*_*n*_^(*i*)^ in each case *i*, *i* = 1,2, 3 [10]. The energy consumption in sleep mode can be easily obtained by

(25) $$\begin{array}{c} E_{S_{16}}=\overset{\infty }{\underset{n=1}{\sum }}\left(E_{n}^{\left(1\right)}p_{n}^{\left(1\right)}+E_{n}^{\left(2\right)}p_{n}^{\left(2\right)}+E_{n}^{\left(3\right)}p_{n}^{\left(3\right)}\right), \end{array}$$

where *E*_*n*_^(1)^ = {[(*Z*_*n*−1_ + 1/*λ*_*u*_)(1 − *e* ^−*λ*_*u*_*T*_*n*_^) − *T*_*n*_ *e* ^−*λ*_*u*_*T*_*n*_^]/(1 − *e* ^−*λ*_*u*_*T*_*n*_^)−(*n* − 1)*T*_*L*_}*E*_*S*_ + (*n* − 1)*T*_*L*_ *E*_*L*_ and its appearing probability is *p*_*n*_^(1)^ = *e* ^−*λZ*_*n*−1_^(1 − *e* ^−*λ*_*u*_*T*_*n*_^) can be found in (8) and (9) in [10]; *E*_*n*_^(2)^ = ∑_*i*=1_^*n*^ *T*_*i*_ *E*_*S*_ + {[(*Z*_*n*_ + 1/*λ*_*u*_)(*e* ^*λ*_*u*_*T*_*L*_^ − 1) − *T*_*L*_ *e* ^*λ*_*u*_*T*_*L*_^]/(*e* ^*λ*_*u*_*T*_*L*_^ − 1) − ∑_*i*=1_^*n*^ *T*_*i*_}*E*_*L*_ and *p*_*n*_^(2)^ = *e* ^−(*λ*_*u*_+*λ*_*d*_)*Z*_*n*_^(*e* ^*λ*_*u*_*T*_*L*_^ − 1) can be found in (13) and (14) in [10]. In Case 3, Zhang and Fujise considered the *n*th listening interval as a part of the sleep mode. However, the *n*th listening interval belongs to the *n*th sleep window and cannot be counted. Therefore, we emend the derivation of *E*_*n*_^(3)^ as

(26) $$\begin{array}{c} E_{n}^{\left(3\right)}=\overset{n}{\underset{i=1}{\sum }}T_{i}E_{S}+\left(n-1\right)T_{L}E_{L} \end{array}$$

and its appearing probability as

(27) $$\begin{array}{c} p_{n}^{\left(3\right)}=e^{-\lambda Z_{n-1}}e^{-\lambda_{u}T_{n}}\left(1-e^{-\lambda_{d}T_{n}}\right). \end{array}$$

Similarly, to get the entire energy consumption of the IEEE 802.16, let *P*_*S*_16__ denote the probability of staying in the sleep mode of the IEEE 802.16 PSM. Thus, we have

(28) $$\begin{array}{c} P_{S_{16}}=\overset{\infty }{\underset{n=1}{\sum }}\left(p_{n}^{\left(1\right)}+p_{n}^{\left(2\right)}+p_{n}^{\left(3\right)}\right). \end{array}$$

Finally, based on (25) and (28), the average energy consumption of the IEEE 802.16 mechanism per unit time can be obtained by

(29) $$\begin{array}{c} \bar{E_{16}}=\left(1-P_{S_{16}}\right)E_{A}+E_{S_{16}}. \end{array}$$

## 5. Numerical Experiment

To validate the performance of DUAL, an object-oriented C++ based energy simulation program has been developed to verify the energy consumption analysis of DUAL. For the reason of more clear comparison with the performance of DUAL, we also adopt the legacy IEEE 802.16 PSM as the control in the simulation. The simulation model is developed based on all system assumptions presented in Sections 2, 3, and 4. The packet arrival rates to DL and UL follow the Poisson process with mean rates *λ*_*u*_ and *λ*_*d*_. To focus on the efficiency of the PSM, we assume that MSs can obtain the needed bandwidth for data transmission once they wake up from the sleep mode. The system parameters and configurations follow the setting of [10] for comparison; for example, *E*_*S*_ = 1, *E*_*L*_ = 10, *E*_*A*_ = 10, *T*_*L*_ = 1, and *Q*_*T*_/*L* = 1600, where *Q*_*T*_ = 1 Mbytes and *L* = 625 bytes [11], throughout all comparisons. The energy consumption is normalized by letting the energy consumption per frame in sleep mode be *E*_*S*_ = 1 and letting the energy consumption per frame in active mode be *E*_*A*_ = 10. Other used simulation parameters are listed in Table 1.

### 5.1. Performance Validation

The generated packet length in the simulation tool follows the exponential distribution with a mean length of 625 bytes, which is referred to the workload statistical analysis in 2008 provided by the cooperative association for Internet data analysis (CAIDA) for IPv4 and IPv6 data packets [11]. It shows that the Internet packets consist of about 50 bytes dominating 50%, 750 bytes dominating 20%, and 1500 bytes dominating 30% based on the statistical results. Other system parameters are set as follows: the initial sleep window *T*_min_ = 4, the listening window *T*_*L*_ = 1, the number of states in sleep mode *m* = 8, and the idle time duration for entering the sleep mode *T*_0_ = 8.

**Figure 4:**
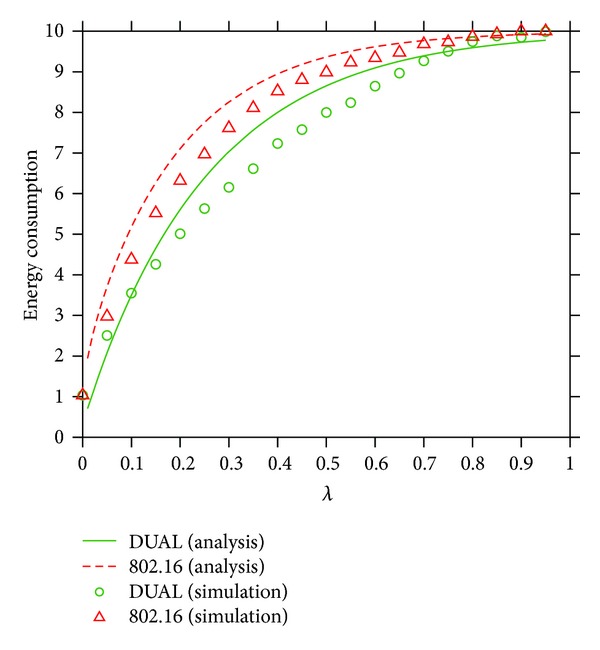
The average energy consumption of DUAL and the legacy IEEE 802.16 in terms of *λ*, where *λ*_*u*_/*λ*_*d*_ = 4/1.

Figure 4 plots the numerical results and simulation results of both $\bar{E_{D}}$ and $\bar{E_{16}}$. Each simulation result is averaged by 10,000 times of independent simulation. The solid and dashed lines are the numerical results obtained from our derived formulae. We can see from Figure 4 that the simulation results are close to the numerical results. The results show that the total energy consumption of DUAL and the legacy IEEE 802.16 is valid. Simulation results also show that DUAL can save more 21.43% energy at most as compared with the IEEE 802.16 PSM when *λ* = 0.3. This observation reveals that DUAL will show its effect on PS when the traffic load is moderate because DUAL has more opportunities to align UL with DL connections. The results also validate the explanation when *λ* ≥ 0.7 because there is no space for DUAL to improve.

### 5.2. Numerical Results

**Figure 5:**
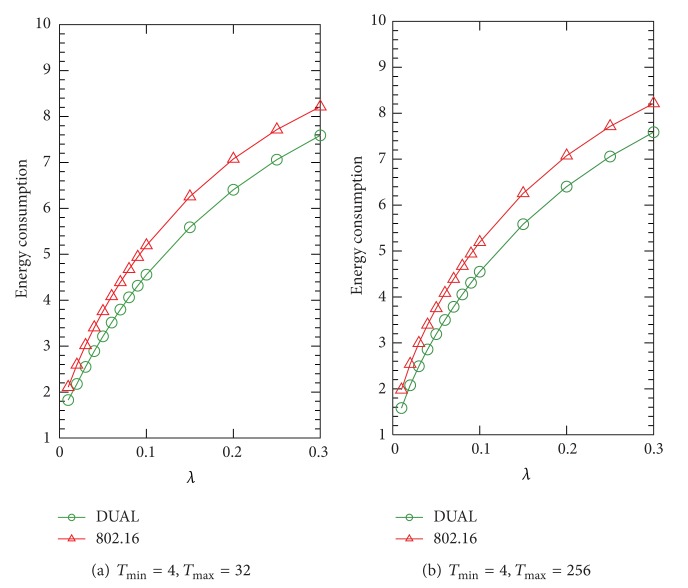
Average energy consumption in terms of *λ*, where *λ*_*u*_/*λ*_*d*_ = 1/4.

**Figure 6:**
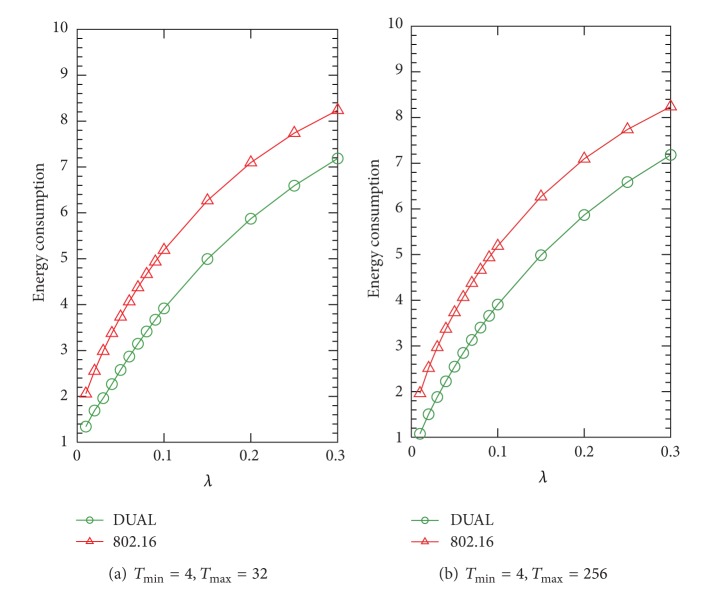
Average energy consumption in terms of *λ*, where *λ*_*u*_/*λ*_*d*_ = 1/1.

**Figure 7:**
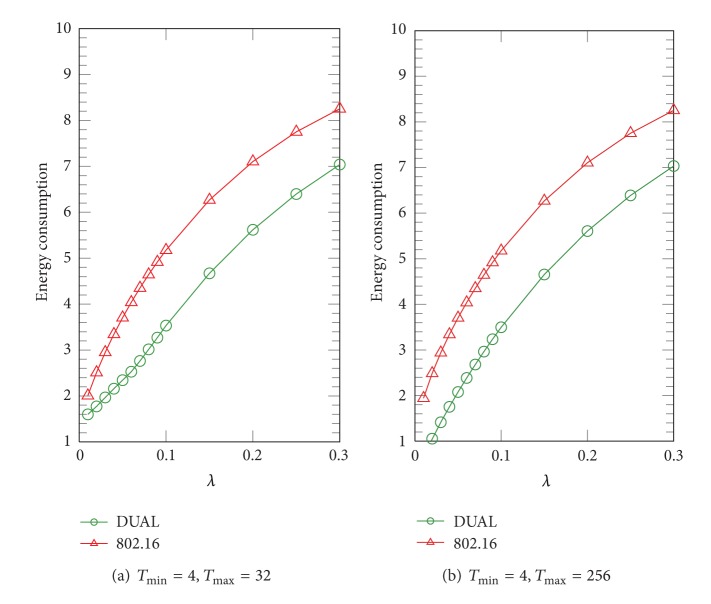
Average energy consumption in terms of *λ*, where *λ*_*u*_/*λ*_*d*_ = 4/1.

In this subsection, we illustrate the average energy consumption per frame of IEEE 802.16 and DUAL PSMs by numerical analysis in different arrival rates *λ*. In Figures 5, 6, and 7, we present a series of experiments in different ratios of *λ*_*u*_/*λ*_*d*_ on the effects of energy consumption. In each figure, we use four different parameters of *T*_max_ to show the effect of the maximal value of sleep window size on the energy consumption. From Figure 5, we can see that the effect of the *T*_max_ value on the energy consumption is not obvious. We also discover that the PS (the gap between the IEEE 802.16 and DUAL) is not significant under the condition of *λ*_*u*_/*λ*_*d*_ = 1/4 as compared with the results of Figure 6. This result indicates that the PS is improved when the DL traffic decreases while UL traffic increases. We can see that the improvement of PS is from 8.57% (*λ*_*u*_/*λ*_*d*_ = 1/4) to 15.71% (*λ*_*u*_/*λ*_*d*_ = 1/1) when *λ* = 0.2.

In Figure 7, we can see a higher improvement of PS under the condition of *λ*_*u*_/*λ*_*d*_ = 4/1. The numerical results show that the improvement of PS is 21.43% when *λ* = 0.2 which matches the result obtained from simulation when *λ* = 0.3 as shown in the last subsection. Intuitively, the MS has higher probability to extend its sleep cycle when the UL arrival rate is higher than the DL arrival rate. This kind of traffic model can be found in the security cameras set up on the side of road or machine to machine (M2M) sensing applications. These cameras or sensing nodes continuously send data back to a remote, centralized server in one direction via wireless communications as well as need a lot of bandwidth to transfer data. In this case, DUAL will benefit this kind of applications.

**Figure 8:**
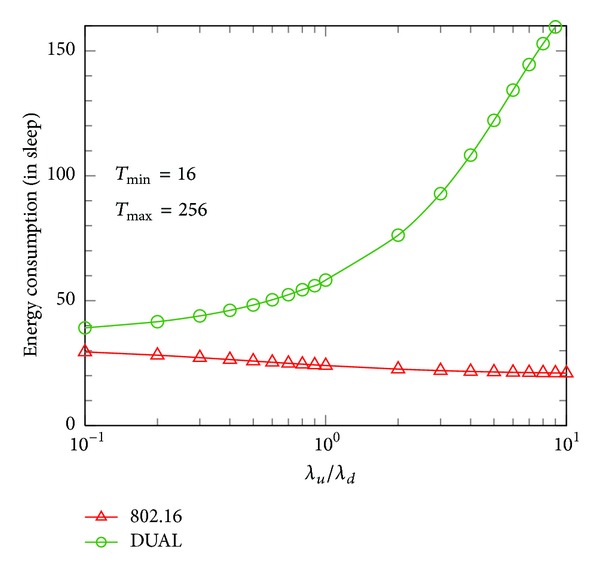
Energy consumption in sleep mode in terms of *λ*_*u*_/*λ*_*d*_ (*λ* = 0.05).

From above comparisons, it is highly interesting to know the impact of different ratios of *λ*_*u*_ to *λ*_*d*_  (*λ*_*u*_/*λ*_*d*_) on energy conservation in sleep mode. For the reason of observing the effect of PS more clearly, we will demonstrate the actual PS in the sleep mode inside.  Figure 8 shows the energy consumption in sleep mode only in terms of different ratios of *λ*_*u*_/*λ*_*d*_ with a fixed *λ* = 0.05. We notice that the energy consumption shown in Figure 8 is the energy consumed in the sleep mode. In other words, the more energy the MS consumes in the sleep mode, the longer duration the MS will stay in the sleep mode. Therefore, we can say that the MS saves more energy if it gets more energy consumption in the sleep mode.  Figure 8 shows that the energy consumed in the sleep mode of the IEEE 802.16 decreases slightly as the ratio of *λ*_*u*_/*λ*_*d*_ increases. Because the IEEE 802.16 protocol only considers the DL traffic transmission to determine the PS duration, the real PS duration will be much shorter than expected value when the UL traffic increases. In contrast, as shown in Figure 8, DUAL shows its capability of staying in the sleep mode (the increasing of energy consumption in the sleep mode) when the ratio of *λ*_*u*_/*λ*_*d*_ increases. The gap between the energy consumption of DUAL (higher) and the IEEE 802.16 (lower) reaches 143.7 when *λ*_*u*_/*λ*_*d*_ = 10. This result shows that DUAL can get longer duration in sleep mode as compared with the legacy IEEE 802.16 when *λ*_*u*_ > *λ*_*d*_. It also shows a fact that DUAL still improves the PS (gets higher energy consumption in sleep mode) when *λ*_*u*_ < *λ*_*d*_.

**Figure 9:**
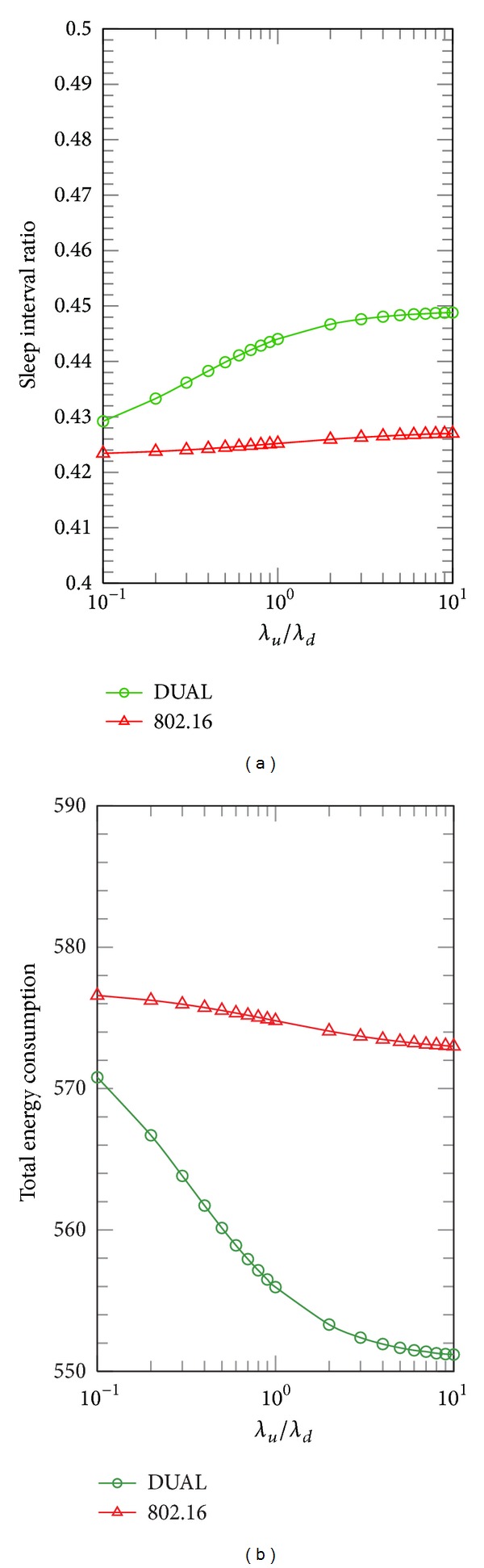
Comparisons of UL traffic impact on sleep interval ratio and total energy consumption for DUAL and the legacy IEEE 802.16 in terms of *λ*_*u*_/*λ*_*d*_ (*λ* = 0.05).

Figure 9 illustrates the impact of UL traffic on total energy consumption if PSMs do not consider synchronizing the DL and UL transmissions. The simulation runs for 100 frames to observe the sleep interval ratio (i.e., the number of frames in sleep mode over 100 frames). Compared with IEEE 802.16 PSM, Figure 9(a) shows that DUAL increases the sleep interval ratio as the UL traffic percentage increases (i.e., *λ*_*u*_/*λ*_*d*_ increases in a fixed packet arrival rate *λ*). This result reveals that DUAL efficiently squeezes more sleep intervals by aligning unsynchronized UL transmission intervals with DL intervals. This result becomes more obvious when the portion of UL traffic becomes higher.  Figure 9(b) shows the total energy consumption during the simulation duration. This result shows that the trend of energy consumption achieved by DUAL decreases as *λ*_*u*_ increases. The gap between the IEEE 802.16 PSM and DUAL becomes slower when *λ*_*u*_/*λ*_*d*_ > 1. This is because the effect of DL and UL alignment becomes lower when the portion of DL traffic is smaller than UL traffic. Finally, it is noticeable that the effect of energy conservation by using DUAL will be more obvious when the energy consumption in active mode *E*_*A*_ is much higher than that in sleep mode *E*_*S*_ (e.g., *E*_*A*_ = 100*E*_*S*_).

## 6. Conclusion

The impact of aligning UL and DL transmissions on energy conservation is investigated in this paper. The energy can be saved further when DL and UL transmissions are overlapped in same transmission periods. According to the discovery, based on the real condition of a limited buffer size *Q*_*T*_ of each mobile equipment, we propose the novel DUAL scheme to align these two opposite connections for getting as many *effective sleep intervals* as possible. Numerical experiments show that DUAL improves the efficiency of energy conservation than the PSM of the legacy IEEE 802.16 protocol. The effect becomes more obvious when the total traffic is light or the ratio of *λ*_*u*_/*λ*_*d*_ > 1. Simulation results and Numerical results are validated and show the efficiency of DUAL. Finally, the experiment results show that DUAL specially suits applications which need a lot of UL bandwidth than DL bandwidth (e.g., security cameras or sensing nodes). These applications meet the trend of future mobile communication applications.

## Figures and Tables

**Table 1:** Parameter in simulation model.

Parameter	Value
The frame length	5 ms
DL/UL ratio	8 : 2
*T*_min_	4
*T*_max_	512
Packet size	625 Bytes
Each MS's buffer size	2000 packets
